# 3D Ultrastructure of the Cochlear Outer Hair Cell Lateral Wall Revealed By Electron Tomography

**DOI:** 10.3389/fncel.2019.00560

**Published:** 2019-12-20

**Authors:** William Jeffrey Triffo, Hildur Palsdottir, Junha Song, David Gene Morgan, Kent L. McDonald, Manfred Auer, Robert M. Raphael

**Affiliations:** ^1^Molecular Biophysics and Integrated Bioimaging Division, Lawrence Berkeley National Laboratory, Berkeley, CA, United States; ^2^Department of Bioengineering, George R. Brown School of Engineering, Rice University, Houston, TX, United States; ^3^Department of Radiology, Geisinger, Danville, PA, United States; ^4^Interdisciplinary Center for Electron Microscopy, University of California, Davis, Davis, CA, United States; ^5^Electron Microscope Laboratory, University of California, Berkeley, Berkeley, CA, United States

**Keywords:** outer hair cell (OHC), subsurface cisternae, cortical cytoskeleton, high pressure freezing and freeze substitution, electron tomography (ET)

## Abstract

Outer Hair Cells (OHCs) in the mammalian cochlea display a unique type of voltage-induced mechanical movement termed electromotility, which amplifies auditory signals and contributes to the sensitivity and frequency selectivity of mammalian hearing. Electromotility occurs in the OHC lateral wall, but it is not fully understood how the supramolecular architecture of the lateral wall enables this unique form of cellular motility. Employing electron tomography of high-pressure frozen and freeze-substituted OHCs, we visualized the 3D structure and organization of the membrane and cytoskeletal components of the OHC lateral wall. The subsurface cisterna (SSC) is a highly prominent feature, and we report that the SSC membranes and lumen possess hexagonally ordered arrays of particles. We also find the SSC is tightly connected to adjacent actin filaments by short filamentous protein connections. Pillar proteins that join the plasma membrane to the cytoskeleton appear as variable structures considerably thinner than actin filaments and significantly more flexible than actin-SSC links. The structurally rich organization and rigidity of the SSC coupled with apparently weaker mechanical connections between the plasma membrane (PM) and cytoskeleton reveal that the membrane-cytoskeletal architecture of the OHC lateral wall is more complex than previously appreciated. These observations are important for our understanding of OHC mechanics and need to be considered in computational models of OHC electromotility that incorporate subcellular features.

## Introduction

Sound is processed in the mammalian cochlea by two distinct types of auditory hair cells located in the organ of Corti: inner hair cells and outer hair cells (OHCs). Inner hair cells transduce sound-induced vibrations into neural signals, while OHCs serve to amplify these vibrations and enhance auditory sensitivity and frequency selectivity (Ashmore, 2008; Dallos et al., 2008; Hudspeth, 2014; Brownell, 2017). The three rows of cylindrically-shaped OHCs make a major contribution to the passive mechanics of the organ of Corti and undergo active length changes in response to electrical stimulation (Brownell et al., 1985; Kachar et al., 1986). OHC electromotility is elicited by changes in the transmembrane potential that result from hair bundle (HB) displacement, with hyperpolarization causing cell elongation, and depolarization causing cell shortening (Ashmore, 1987; Santos-Sacchi and Dilger, 1988).

The process of electromechanical transduction occurs in the lateral wall of the OHC (Holley and Ashmore, 1988a; Dallos et al., 1991), which extends from just below the cuticular plate at the apex of the cell down to the basally located nucleus. Several early studies provided evidence that electromotility is based in the basolateral plasma membrane (PM; Kalinec et al., 1992; Hallworth et al., 1993; Huang and Santos-Sacchi, 1994), and phenomenological models have been developed to account for experimental observations (He et al., 2006; Spector et al., 2006; Ashmore, 2008). Although the discovery of the transmembrane protein prestin (Zheng et al., 2000) provided a molecular basis for a membrane-based electromechanical transduction process (Liberman et al., 2002; Gao et al., 2007; Dallos et al., 2008; Santos-Sacchi et al., 2017), there are still many unanswered mechanistic questions regarding the relation between prestin activity and cellular length changes. Current models of OHC electromotility posit that a PM-resident motor generates the force required for cell length changes, either through area-change (Dallos et al., 1993; Iwasa, 1994) or nanoscale bending (Raphael et al., 2000). These models inspired research into how molecules that disrupt membrane curvature and mechanics affect OHC function. For example, the non-steroidal anti-inflammatory agent (NSAID) diflunisal was recently shown to increase the length of OHCs by up to 8%, yet the electromotility response remained robust (Duret et al., 2017). The structure of the OHC must, therefore, be able to both accommodate changes in resting cell length and also support voltage-induced motility. Clearly, a full biophysical understanding of OHC mechanics and electromotility requires more detailed knowledge of the 3D sub-cellular and molecular architecture of the OHC lateral wall.

The current structural understanding of the OHC lateral wall represents an amalgamation of results from studies spanning multiple decades, utilizing transmission electron microscopy (TEM), scanning electron microscopy (SEM) and atomic force microscopy. The lateral wall can be viewed as a trilaminate composite (Brownell et al., 2001), consisting of the PM, an underlying cytoskeletal network, and an adjacent system of circumferential lamellar organelles known as the subsurface cisternae (SSC). In freeze-fracture, ~10 nm intramembranous particles were reported in the PM that have been ascribed to the membrane motor protein prestin (Forge, 1991; Kalinec et al., 1992; Santos-Sacchi et al., 1998). The cytoskeletal network lying in the ~30 nm wide extracisternal space (ECS) between the PM and SSC is often referred to as the cortical lattice (CL). It consists of patches of locally parallel, circumferentially oriented filaments of actin connected by axially oriented cross-links, which were assumed to be spectrin polymers (Holley and Ashmore, 1988b, 1990; Forge, 1991; Holley et al., 1992; Wada et al., 2004). This architecture was used to explain observations that the circumferential stiffness of the OHC was greater than its axial stiffness (Tolomeo et al., 1996); the resulting anisotropy in lattice stiffness was purported to direct the energy from an isotropic PM motor down the axis of the cell. A structure spanning the ECS between the PM and actin filaments has been observed and termed the pillar due to its morphology; estimates on pillar width range from 6 to 10 nm (Flock et al., 1986; Forge, 1991; Holley et al., 1992). In mechanical models of electromotility, the pillar is presumed to provide the necessary coupling of forces generated in the membrane to the underlying cytoskeleton to generate whole-cell shape changes (Brownell et al., 2001; Spector et al., 2006).

While the SSC is a prominent sub-structure in the lateral wall, its role in OHC function remains obscure. The SSC closely abuts the CL, and the number of concentric SSC layers varies according to species, the position of the OHC along the cochlea, and longitude along the lateral wall of an individual OHC (Furness and Hackney, 1990; Forge, 1991; Slepecky, 1996). One SSC layer occupies a similar volume as the PM/CL complex; a previous study reports an average cisternal width of 27 nm (Dieler et al., 1991), comparable to the span of the ECS. No study to date has shown direct interaction of the SSC with the PM/CL components underlying the motile mechanism, and thus it has been unclear whether the SSC plays a role in electromotility. The potential importance of the structure and electrical properties of the SSC has been the subject of recent research (Song and Santos-Sacchi, 2015).

Prior to this study, the characterization of the OHC lateral wall by electron microscopy techniques has been limited by sample preservation and by the conventional 2D projection imaging of a 3D structure, resulting in superposition of molecules along the electron path. However, a full understanding of electromotility requires an accurate 3D depiction of the dimensions and arrangement of the molecular constituents present within the OHC lateral wall. This is essential for understanding how the micromechanical 3D architecture gives rise to the passive mechanical properties of the OHC, and to assess whether electromechanical force transmission by prestin in the PM alone is likely to be sufficient to deform the entire OHC lateral wall. To address this problem, we employed high-pressure freezing/freeze-substitution (HPF/FS; McDonald and Auer, 2006) to guarantee high fidelity sample preservation, along with electron tomography for 3D imaging of the OHC lateral wall.

Our research has revealed several major new findings. First, both the SSC membrane and its lumenal material (LM) are composed of an intricate and highly organized structure not previously reported in any intracellular organelle. Second, the actin filaments of the CL appear tightly connected to the adjacent SSC membranes by previously unresolved short linkages. Thus, these observations reveal the SSC to be a structurally rich organelle tightly associated with the actin cytoskeleton. In contrast, the pillar proteins that connect the PM to the cytoskeleton are thinner and significantly more variable than previously observed. Overall, these ultra-structural findings significantly advance our understanding of the subcellular architecture of the lateral wall and provide insight into how this architecture contributes to the overall mechanical properties of the cochlear OHC.

## Materials and Methods

### Preparation of Samples

#### HPF of OHCs

Hartley albino guinea pigs were anesthetized by isoflurane and decapitated with a guillotine. The temporal bones were removed and the organ of Corti was exposed by dissection in Invitrogen Medium 199 containing Hanks’ salts (Invitrogen Corporation, Carlsbad, CA, USA). Strips of the sensory epithelium were transferred using a 200 μl pipette and placed in a 35 mm MatTek dish (MatTek Corporation, Ashland, MA, USA); enzymatic digestion was avoided to preserve long strips of tissue. In brief, long strips containing OHCs were then identified by stereoscope and selected for further manipulation/handling using a microaspirator connected to 200 μm inner diameter Spectrapor dialysis tubing with an MW cutoff of 13–18 kDa (Spectrum Laboratories Inc., Rancho Dominguez, CA, USA). Using the microaspirator, samples were transferred to 10% glycerol (v/v), 20% dextran (w/v), or 20% BSA (w/v) as filler material and drawn back into the dialysis tubing. The tubing was then crimped to enclose the sample, transferred to either a 150 or 200 μm deep aluminum hat, the remaining space in the hat was filled with the same filler material, and the sample was cryoimmobilized using a BAL-TEC HPM-010 high-pressure freezer (BAL-TEC, Inc., Carlsbad, CA, USA). Details of this procedure and the apparatus used are found in Triffo et al. (2008). This study was carried out in accordance with the recommendations of the AVMA Guidelines for the Euthanasia of Animals^1^. The animal protocol was approved by the Lawrence Berkeley National Laboratory Animal Welfare and Research Committee (AWRC).

#### Freeze-Substitution

Samples were freeze-substituted in 1% osmium tetroxide and 0.1% uranyl acetate in acetone using a Leica AFS (Leica Microsystems, Vienna, Austria) following a previously described protocol (McDonald, 2007). In some samples, 1–2% of water was added to enhance membrane contrast (Walther and Ziegler, 2002). Following freeze substitution, specimens were washed with several rinses of pure acetone before being infiltrated in either an Epon-Araldite mixture (McDonald and Müller-Reichert, 2002) or Durcupan ACM (Electron Microscopy Sciences, Hatfield, PA, USA). The tubes were flat-embedded between two slides using two layers of parafilm as a spacer (Müller-Reichert et al., 2003).

#### Section Preparation

Flat-embedded tubes were screened, mounted on blank epoxy blocks for sectioning, and their orientation for sectioning recorded using an Olympus SZX12 stereoscope (Olympus America, Inc., Center Valley, PA, USA) with epi- and trans-illumination provided by a pair of Fostec 150W lamps (Olympus). One-hundred and fifty nanometer sections were collected on formvar coated slot grids using a Reichert Ultracut E ultramicrotome (Leica). The sections were post-stained using a sequence of either 2% uranyl acetate in 70% methanol followed by Sato’s lead citrate (Sato, 1968), or 2% KMnO_4_ followed by a PALS bleach step and Sato’s lead citrate. Five or 10 nanometer gold fiducials were added to both sides by floating on a drop of colloidal gold (British BioCell International, Cardiff, UK) following pre-treatment of each side with 0.001% (w/v) poly-L-lysine in double-distilled water.

### Electron Tomography

All data presented represent dual-axis tilt series taken by rotation about orthogonal axes. Tilt series of 150 nm sections were collected at 1° increments through a range of ±70° using EMMENU 3.0 software (TVIPS GmbH, Gauting, Germany) on a Phillips CM200 TEM (FEI, Eindhoven, Netherlands) equipped with a Fischione Advanced Tomography Holder (E. A. Fischione Instruments, Inc., Export, PA, USA) and a Tietz TEMCam-F214 2k × 2k CCD camera (TVIPS), or using SerialEM (Mastronarde, 2005) on a JEOL JEM-2100F (JEOL Limited, Tokyo, Japan) equipped with a Tietz TEMCam-F415 4k × 4k CCD (TVIPS). Images were collected with an ~8 Angstrom pixel size at 1–1.5 μm defocus to preserve ~20 Angstrom information in the raw data, as evidenced by Thon ring position in resulting micrograph power spectra. Series were aligned with 5 or 10 nm gold fiducials and reconstructed using weighted back-projection with the IMOD software package (Kremer et al., 1996).

### Post-processing and Segmentation

Post-processing and segmentation of the resulting volume density maps was done using a combination of IMOD and the UCSF Chimera package (Pettersen et al., 2004; Goddard et al., 2007). Density maps were noise-filtered using the non-linear anisotropic diffusion (NAD) filter in IMOD and inspected in non-orthogonal orientations using the slicer tool. Line profiles (see Supplementary Figure S1) across linear objects reveal that the signal is clearly above the noise level, in accordance with the fact that linker molecules can be readily detected.

Features of interest such as the actin filaments, PM, SSC membrane and LM, as well as the pillars and actin-SSC links were visually detected. Interpretation of structure density can be complicated by the presence of other proteins which reside in the immediate vicinity of such structures, and semiautomated segmentation approaches such as the super-pixel segmentation feature of the ORS Dragonfly software can assist with feature extraction (see Supplementary Figure S2). To avoid overinterpretation of fine features within the density maps, we utilized simplified ball-and-stick models superimposed on the density maps for analysis, rather than volume segmentation of individual features. This also has the advantage that the geometrical properties of the model (such as length, thickness, and spacing) can be easily measured (see Supplementary Figure S2). For measurements of the pillar and actin-SSC link spacing along the actin filament, a resolution threshold of 4 nm was used, which resulted in the exclusion of one pillar spacing measurement.

## Results

### Advantages of High Pressure Freezing/Freeze Substitution and Electron Tomography

Multiple preparation protocols were evaluated using both dissected organ of Corti along with decalcified intact cochleae. These included conventional preparation protocols using paraformaldehyde/glutaraldehyde followed by osmium tetroxide containing potassium ferricyanide, progressive lowering of temperature (PLT) dehydration prior to resin embedding, and HPF followed by FS prior to resin embedding. All data presented here are derived from HPF/FS applied to rapidly dissected strips of guinea pig OHCs as described previously (Triffo et al., 2008), as such samples displayed the least extraction and best preservation of cytosolic features as judged by cytoskeletal and SSC details. Over the course of this project, 16 guinea pigs were sacrificed to optimize sample preparation and obtain data; nine guinea pigs produced 75 HPF samples that yielded approximately 50 FS specimens that were screened for optimal quality to undergo tomographic analysis. In total, 13 dual-axis tomograms of the guinea pig OHC lateral wall were reconstructed and analyzed for this study, obtained from four animals. The data reported in this article was derived from the best three tomograms, obtained from two animals.

**Figure 1:**
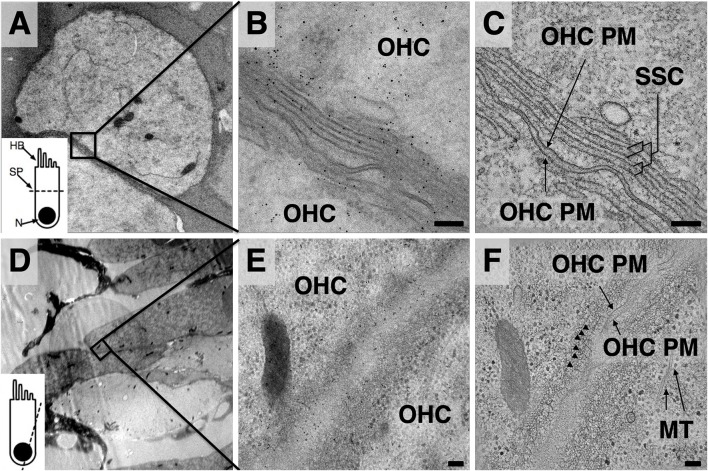
Comparison of projection images and z-planes from outer hair cell (OHC) lateral wall tomograms. **(A,D)** Low magnification overviews. Inset depicts orientation of plane of sectioning—**(A)** axial; **(D)** closer to longitudinal. HB, hair bundle; SP, section plane; N, nucleus. **(B,E)** Projection images from region indicated in black boxes of **(A,D)**. **(C,F)** Mid-volume z-planes from the resulting tomograms. Arrowheads in **(F)** indicate individual actin filaments in the Cortical lattice (CL). MT, microtubules. Scale bars = 100 nm.

Figure 1 illustrates two representative regions in different orientations that were selected for tilt-series acquisition; the top row is from an axial cut through an OHC, while the bottom row is from an oblique angle closer to a longitudinal section. The top sample was prepared with 20% BSA as a filler during HPF (appearing as dark contrast outside the cell); the bottom sample was prepared using 20% dextran as filler. Dextran was optically transparent after embedding, allowing for easier sample preparation and orientation; however, these samples are more difficult to section. The samples using 10% glycerol as filler yielded optical transparency along with good sectioning properties, but in the case of guinea pig OHCs, the osmotic load resulted in the collapse of the cells. During isolation of OHC strips for HPF, OHCs often come into close proximity with each other; this allowed data acquisition from regions where two cells’ worth of lateral wall could be imaged in the same tomogram to maximize the area surveyed.

The unique advantage of applying electron tomography over conventional 2D projection TEM imaging to the study of OHC lateral wall components is demonstrated by comparison of projection (Figures 1B,E) and extracted z-planes from the resulting tomograms (Figures 1C,F), revealing the improved resolution in the z-direction of the reconstructions. In Figure 1C, internal membranes that delineate the boundaries of each SSC cisterna are sharply defined, allowing precise mapping of changes in curvature and continuity in the outermost cisterna. In Figure 1F, circumferential actin filaments that are nearly parallel to the z-plane are clearly visible just beneath the PM (marked by arrowheads), whereas in projection views alone these filaments are obscured (Figure 1E). This illustrates the advantage of electron tomography compared to projection TEM, along with the challenges of image analysis in a crowded, non-extracted cellular environment.

### Structure of the SSC

**Figure 2:**
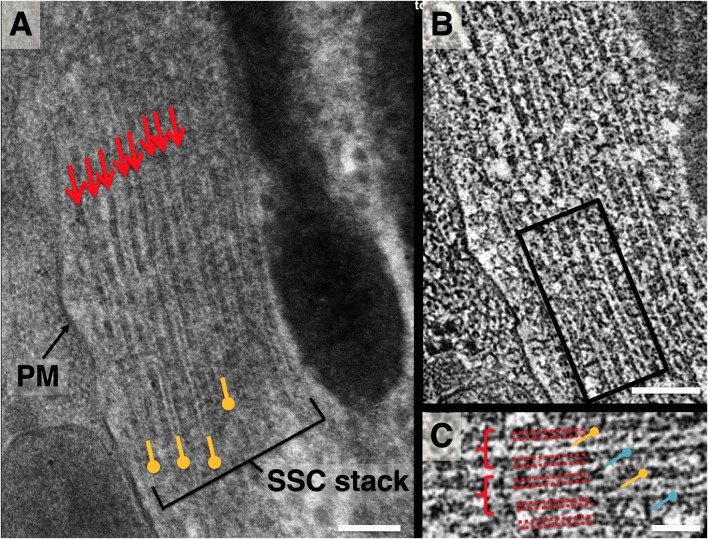
Lateral view of subsurface cisterna (SSC) structure. **(A)** Close-up projection view of lateral wall in longitudinal section. Small arrows indicate membranes of SSC cisterna; pushpin annotation denotes lumenal material (LM); **(B)** 4 nm averaged z-plane from a tomogram of **(A)**; **(C)** close-up of two cisternae from the box in **(B)**, denoted by dotted red lines, showing punctate cross-section of LM (yellow pushpin), occasional contacts between LM and adjacent SSC membrane, and cisterna-cisterna connections (blue pushpin). Scale bar in **(A,B)** = 100 nm, **(C)** = 50 nm.

In projection, each individual subsurface cisterna appears as a 3-layer “sandwich” consisting of two boundary membranes plus a band of lumenal material (LM), as shown in Figure 2. The LM occupies approximately a third of the central lumen volume between the outer and inner cisternal membranes. This observation was consistent across multiple samples from several freezing sessions with varying HPF filler material concentrations (20% BSA, 20% Dextran, w/v) and FS protocols. When arranged in stacks, a sequence of thick and thin bands is routinely observed, corresponding to SSC membrane and LM, respectively (Figure 2A).

Z-sections from a tomogram of this stack (Figures 2B,C) reveal discrete density between adjacent cisternae, implying distinct connections between the membranes of neighboring cisternae. In each lumen, the LM appears as a combination of both continuous density and discrete punctae. The individual SSC membranes are of uniform density and appear at least as thick as the PM (Figure 1C).

**Figure 3:**
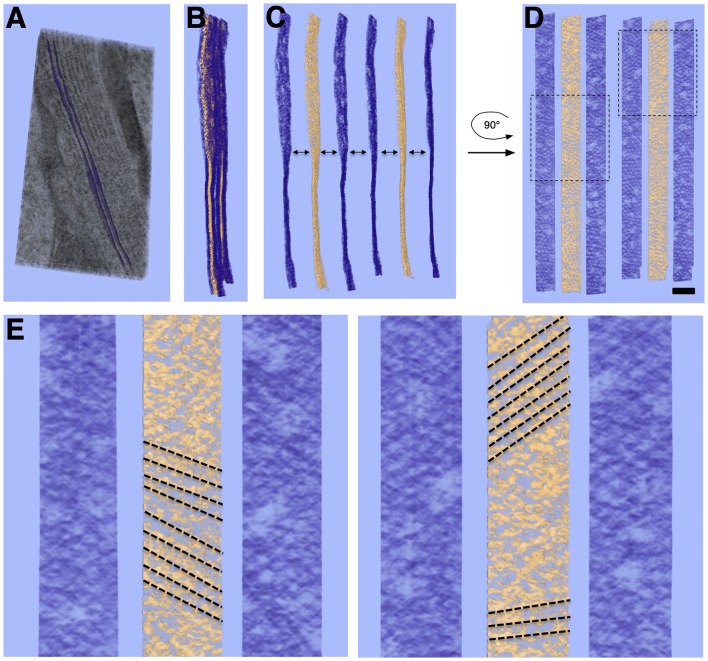
Volume rendering and en-face view of SSC membrane pattern and LM. **(A)** Volume rendering of tomogram slab with two membranes delineating an SSC cisterna rendered as purple surfaces. **(B)** Two cisterna density maps extracted from **(A)** using such surfaces; membrane density is rendered in purple, with LM rendered in gold. **(C,D)** Separation of membrane and LM along with 90° rotation along the y-axis to yield en-face views of the membrane and LM, revealing local hexagonal membrane pattern and patch-like linear organization of LM material. **(E)** Close-up of two regions from **(D)** indicated by dotted-line boxes. Linear organization of LM, indicated by dotted-line annotation, can also be seen in regions of the volume rendering. The directionality of the LM organization appears to coincide with the lattice lines of the honeycomb pattern in the adjacent SSC membranes. Scale bar in **(D)** = 100 nm, **(E)** = 50 nm.

**Figure 4:**
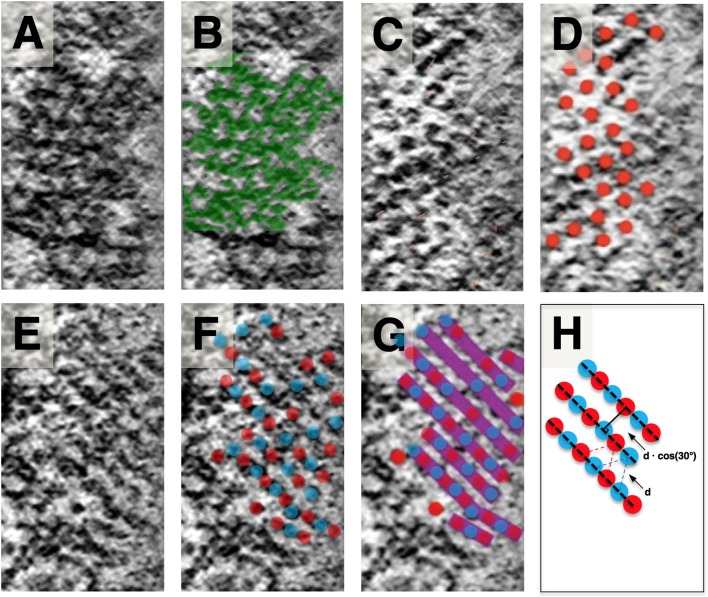
Slice planes through the SSC LM and comparison to local SSC membrane pattern. Images are organized in horizontal pairs, with annotation of density superimposed to the left of identical tomogram image. **(A,B)** SSC honeycomb pattern observed at the outermost face for reference. **(C,D)** LM appears as discrete disks. **(E)** LM appears as continuous bands (most common observation). **(F,G)** Schematic overlay on **(E)**, illustrating our interpretation of the bands consisting of two interdigitating groups of hexagonally arranged disks. Disks are colored red and blue to reflect that the hexagonally arranged disks are associated with the two different SSC membranes of each cisterna. **(H)** Because of the apparent underlying hexagonal organization, the distance between these bands is related to the hexagonal lattice spacing by cos (30°). Scale bar **(H)** = 25 nm.

A quasi-periodic variation in density is seen within the surface of each SSC membrane, interrupted by sporadic defects in the surface (Figures 3, 4A). No similar pattern is observed in the PM. Visual inspection reveals a “honeycomb” pattern of the SSC membrane density, with the density arranged along the edges of a hexagonal grid. Considering the local organization to be a hexagonal lattice and measuring along the edges of the resulting equilateral triangles, the average spacing between unit cells is 19.2 ± 1.9 nm (*n* = 137, using planar patches derived from a single tomogram acquisition). Due to feature size, even gentle curvature of the SSC membrane surface prevents capturing large stretches (more than ~100 nm) of the pattern within a single interpolated plane. Thus, surfaces that were modeled using plane-by-plane contours were used to extract volume regions enclosing each SSC membrane for volume rendering. This sequence, along with an en-face rendering of the SSC membrane pattern, is illustrated in Figure 3.

When viewed en-face, the LM is most commonly organized in rod-like parallel rows (Figure 4), with rows of density aligning with the lattice direction of the adjacent SSC pattern (Figures 3D,E). Occasionally this row pattern is absent, and groups of individual disk-like punctae, approximately 10 nm in diameter, are seen within the LM (Figure 4). The disks are arranged in a quasi-hexagonal pattern with a spacing of 19.1 ± 2.1 nm (*n* = 64; where *n* reflects discrete disks resolved in planar patches derived from a single tomogram acquisition). The central LM makes contact with its adjacent SSC membranes (Figures 2B,C), linking together the two membranes of an individual cisterna. We have interpreted the row pattern as two interdigitating quasi-hexagonal lattices, proposing that interdigitating groups of hexagonally patterned LM disks associated with opposing membranes in an SSC give rise to these continuous bands of density, which we have depicted blue and red (Figures 4F,G). In a hexagonal lattice, the orthogonal distance *d* between lattice lines is related to the lattice spacing *a* by the relationship *d = a* * cos(30°); for *a* = 19.1 ± 2.1 nm, *d =* 16.5 ± 1.8 nm. When organized as rows, the orthogonal distance between adjacent rows is measured to be 16.8 ± 1.3 nm (*n* = 61, using planar patches derived from a single tomogram acquisition), in excellent agreement with the expected value calculated from the lattice spacing of the individual LM disks.

### Morphology of the SSC and Plasma Membrane

**Figure 5:**
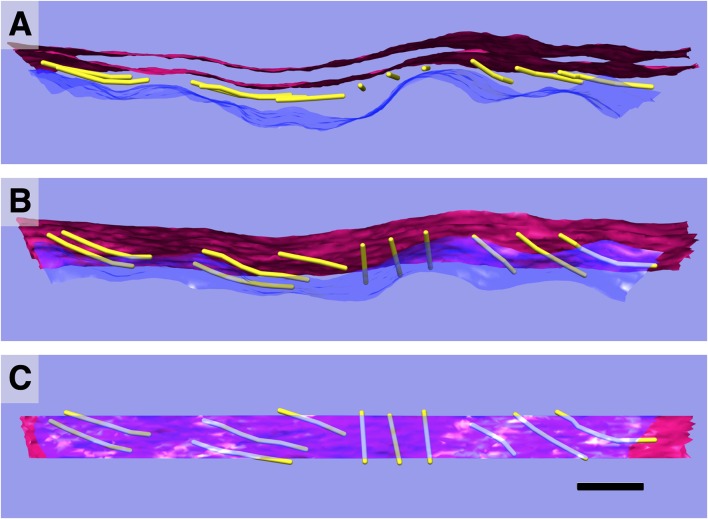
3D relationships of plasma membrane (PM), actin, and SSC. Model of single SSC cisterna, actin, and PM **(A)**; rotated 45° **(B)**; and 90° **(C)** about the y-axis. PM in blue/translucent blue; SSC membranes in red, and actin rendered as 8 nm diameter gold filaments. Scale bar = 100 nm.

In our isolated guinea pig OHCs, the outermost layer of the SSC is largely continuous rather than fenestrated, which is often observed by others under conventional preparation methods (Furness and Hackney, 1990; Dieler et al., 1991; Slepecky and Ligotti, 1992). The width of each cisterna is uniform, consistently measuring 28–30 nm between the center of each membrane. When in layers, the individual cisternae are close together but do not touch, leaving a gap typically less than 20 nm that is bridged by cisterna-cisterna connections. The dextran and BSA fillers used to prevent ice formation during the freezing process contributed ~50 mOsm to the dissecting media, which may explain the undulating appearance of the PM. Alternatively, we should not discard the possibility that the curvature we have observed in the PM is a true feature in OHCs, in accordance with an early TEM study of OHCs (Ulfendahl and Slepecky, 1988). The PM of many cell types shows deviations from a perfectly straight or round phenotype, which constitutes a “membrane reservoir.” The existence of a membrane reservoir in the OHC lateral wall has been demonstrated by pipette aspiration experiments (Morimoto et al., 2002), fluorescence polarization microscopy (Greeson and Raphael, 2009) and TEM observations (Ulfendahl and Slepecky, 1988). Interestingly, the SSC consistently maintains a smooth appearance despite these variations in PM curvature. This is illustrated in Figure 5A, where the extracted surfaces representing the outermost SSC layer and PM are rendered with PM in light blue and the SSC membranes in red.

### Actin Is Physically Connected to the PM and the SSC

**Figure 6:**
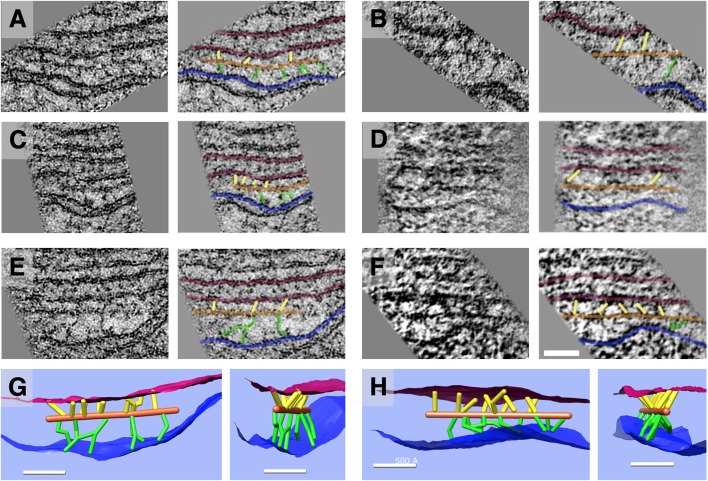
Slice planes and model renderings from tomograms at orientations capturing actin-membrane links. **(A–F)** In the top three rows, six separate actin filaments are depicted; each image has an identical copy to its right with an overlay of the ball-and-stick model in the following colors: red = SSC membrane; blue = PM; orange = actin; green = pillar; yellow = actin-SSC link. **(G,H)** In the bottom row, renderings of 3D models corresponding to the filament complexes in the third row **(E,F)** are oriented (side view and axial view) to illustrate the distribution of actin-membrane links not apparent in single 2D slice views. Scale bar = 50 nm.

Individual paths of circumferential actin filaments were segmented out and rendered, revealing 8 nm wide cylinders consistent with anticipated actin filament dimensions (Figure 5). The filaments appear to be organized in domains, consistent with the “domain” pattern observed previously (Holley et al., 1992; Wada et al., 2004), with short patches of several filaments running parallel to one another. Furthermore, thin connections between the actin filaments and the PM are also evident, and likely correspond to the pillar protein described in previous studies (Flock et al., 1986; Forge, 1991; Holley et al., 1992; Figure 6). However, unlike the images depicted in previous projection TEM studies, pillars were found to be commonly less than half as wide as adjacent actin, with regions of individual pillars frequently as thin as 3–4 nm. Of all the components of the lateral wall—PM, CL, SSC, and their associated connections—these pillar connections between PM and actin exhibited the highest variability in morphology. They range in length from 17 nm to 51 nm with an average of 28 nm ± 11 nm (*n* = 21). Their average spacing along the actin filament was 26 nm ± 11 nm, ranging from 6 nm to 43 nm (*n* = 15). When compared to the adjacent SSC and PM, the actin filaments maintain a consistent spacing from the SSC membrane even when an undulating PM is observed (Figure 5). Slice planes through multiple tomographic volumes reveal discrete connections between the actin and SSC membrane, which we refer to as actin-SSC links (Figure 6). They range in length from 13 nm to 29 nm with an average of 19 nm ± 5 nm (*n* = 24). Their average spacing along the actin filament was 20 nm ± 11 nm, ranging from 5 nm to 42 nm (*n* = 17). There is no previous report of an actin-SSC linkage, and their existence establishes mechanical coupling between the actin and outermost SSC. Combined with the thinner than expected pillar connections between the PM and actin, the actin-SSC links complete the physical connection between PM and SSC. It is interesting that in cases of undulating PMs, the pillar structures are found to vary in length whereas the actin-SSC links remain relatively constant.

## Discussion

### SSC Membrane and Lumen Substructure

**Figure 7:**
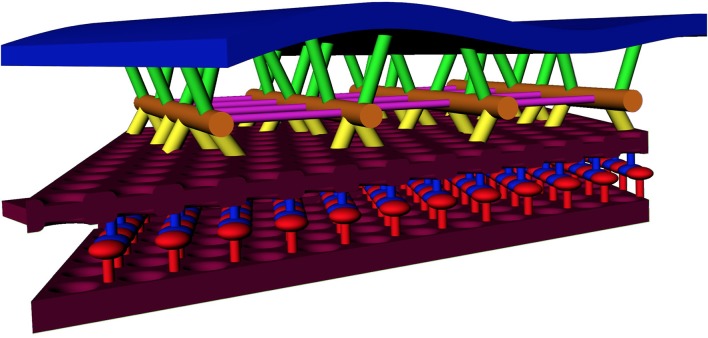
Model of the OHC lateral wall, including new SSC structure and actin-SSC links. Annotation: blue = PM; maroon = SSC membrane; orange = actin filament; yellow = actin-SSC link; green = pillar. For the SSC lumen material, red and blue are used to indicate the alternating material associated with each SSC membrane (see Figure 4). Cross-links between actin, shown in other studies, are colored purple.

Our observations on the structure of the SSC membrane and LM are summarized in Figures 2–4. Visual inspection of these figures reveals an apparent hexagonal pattern within the SSC membrane, with distortion due to curvature of the membrane limiting characterization of this structure to local patches. While the LM was most often observed as continuous bands of density aligned with the lattice of the adjacent SSC membrane pattern, in regions lacking this striped pattern we see discrete disk-like objects which appear to be hexagonally arranged and regularly spaced (Figure 4); the spacing of these disks matches that of the SSC membrane pattern (~19 nm). In light of these observations, we posit that the stripe pattern in the lumen arises by interdigitating two hexagonal patterns of these discrete disks, which we propose to originate from each of the two membranes of the SSC and which we arbitrarily assigned a red or blue color (Figure 4). The predicted distance between the stripes matches closely the distance between the stripes found in the tomogram, in support of our proposed model. These observations also suggest that the “red” and “blue” densities may attach in the lumen of the SSC, as depicted in our overall proposed model of the OHC lateral wall structure (Figure 7).

The hexagonal structure within the membranes of the SSC displays a level of organization not typically associated with the membranes of similar organelles (ER, SER, SR, Golgi apparatus). To our knowledge, this pattern has not been previously described in the SSC membrane, although there is a report that rosette-shaped particles are associated with the lumenal surface of the SSC membrane (Kakehata et al., 2000). However, the pattern we observe is a connected, honeycomb-like structure within the SSC membrane, rather than a field of discrete rosette particles. The repeat length of 19.2 nm and space between the protein densities in the honeycomb-like pattern imply that such a pattern cannot be explained by dense packing of 10–11 nm intramembranous particles that were described for the PM (Forge, 1991; Kalinec et al., 1992; Santos-Sacchi et al., 1998). In our samples, we applied the same en-face visualization to the PM density, but could not detect any regular protein pattern within the PM.

While proteins that do not extend beyond the thickness of the membrane generally cannot be differentiated from the lipid components of membranes preserved by FS, we found the SSC membrane pattern to be most prominent under FS conditions containing 100% acetone. Such conditions are known to deemphasize internal membrane contrast, presumably by destabilization of phospholipid head groups (Buser and Walther, 2008). This suggests that the honeycomb density we have observed in the SSC correlates to an underlying protein scaffold supporting the structure of the membrane.

Knowledge of molecular constituents of the SSC is sparse. In ascribing function to the SSC, correlates have been drawn to similar structures in other motile cells, such as the sarcoplasmic cisternae of striated muscle fibers, with the postulate that the SSC may serve as a calcium store (Frolenkov et al., 2001; Frolenkov, 2006). In this vein, immunogold labeling has provided evidence for the localization of the ryanodine receptor (ryR) to the SSC membrane (Grant et al., 2006). However, the known dimensions of ryR, with a cytosolic domain 24–25 nm wide (Serysheva et al., 2005), are not in agreement with the 19.2 nm repeat we show to be the dominant component of the SSC membrane. Further, ryR has 4-fold symmetry (Serysheva et al., 2005), which is not compatible with a quasi-hexagonal lattice. Thus, the SSC membrane pattern we observe is unlikely to be composed of ryanodine receptors.

The LM present within the SSC is intriguing; we are not aware of other examples of intracellular compartments with such regularly arranged structure in the lumen. This regularity is present in several ways: its location precisely along the mid-plane of the lumen, the hexagonal arrangement of discrete punctae, and its organization into parallel, equidistantly spaced rows. Because the central LM is connected to its opposing membranes, an individual SSC cisterna must be treated as a composite sandwich consisting of its two bounding membranes and the LM. From a structural perspective, the SSC appears more akin to a truss bridge than the “floppy sack” that may be inferred from previous TEM studies that at times have revealed less uniform, empty cisternae likely due to fixation, extraction, and dehydration artifacts typically associated with conventional sample preservation methods.

### Pillar Proteins Provide Thin Plasma Membrane-Cytoskeletal Connections

The wavy appearance of the PM in our preparations may be explained by the osmotic stress imposed by our HPF filler requirements, disrupting the regular spacing of density ascribed to pillar proteins in previous studies (Forge, 1991). In cases of osmotic stress and the resulting PM variations, we find the pillar density joining PM to actin to vary in length and orientation, reflecting the variation in distance between the undulating PM and the actin filaments, whereas the actin filaments appear unaffected by the varying curvature of the PM and the resulting length differences of the pillar proteins, and instead remain tangent to the surface of the SSC membrane. This finding implies that the molecular interactions that connect the SSC to actin are stronger than those that connect the PM to actin. Interestingly, regions of individual pillars are frequently as thin as 3–4 nm and never exceed the cross-sectional area of an actin filament. The dimensions and the variable orientation raise the question of how such a delicate structure would effectively transmit the shear applied by a PM motor to the actin filament and avoid buckling in the process. Combined with the smooth appearance of the SSC membrane, its intermembrane quasi-periodic protein network, and preserved actin-SSC connections, it would seem that the pillar may be the weakest link in the coupling between PM, actin lattice, and SSC. This observation is consistent with micro-aspiration experiments demonstrating the separation of the PM from the SSC-CL complex (Oghalai et al., 1998).

### Implications for OHC Mechanics

A cartoon schematic incorporating our findings into a new model of the lateral wall is illustrated in Figure 7. At a minimum, the substructure visualized in the SSC could explain a cisterna’s ability to maintain the spatial tolerances required of 9–10 μm diameter concentric cylinders of membranes spaced 20–30 nm apart and running the length of the OHC, spanning distances that can exceed 50 μm. Our structural findings are consistent with AFM studies indicating that the OHC possesses a highly elastic cortical shell organization (Zelenskaya et al., 2005). This structure is also likely coupled to the maintenance of OHC turgor pressure, which is required for electromotility (Brownell, 1990; Chertoff and Brownell, 1994). The structural details of the SSC also have relevance for biophysical models of current flow within the cell and development of potential intracellular voltage gradients (Halter et al., 1997; Nygren and Halter, 1999). The revealed molecular organization of the SSC raises the possibility that the SSC layer is mechanically anisotropic. In the radial direction, the LM is densely packed and not likely to easily accommodate strain, whereas, in the longitudinal direction, the relative motion could potentially occur between the rows of densely packed material. Thus, the overall mechanical anisotropy of the OHC lateral wall, previously identified solely with the actin-spectrin cytoskeleton (Tolomeo et al., 1996), may also be reflected in the SSC. Previous results that the number of cisternae can vary depending on the axial position along the OHC lateral wall, location in the cochlear spiral, and animal model (Furness and Hackney, 1990; Lutz and Schweitzer, 1995; Kakehata et al., 2000), also raise the possibility that regulation of SSC structure may contribute to OHC specialization.

In summary, the novel ultra-structural findings presented here arise from a combination of advancements in sample preservation *via* HPF/FS, combined with the ability to resolve 3D structural relationships among components within the lateral wall through the use of electron tomography. The novel structural features observed motivate research to determine the molecular composition of the SSC, the identity of the cytoskeletal cross-linking molecules, and the implications of these findings for the biophysical mechanism of OHC electromotility.

## Data Availability Statement

The datasets generated for this study can be found in wwPDB, EMD-20547.

## Ethics Statement

This study was carried out in accordance with the recommendations of the AVMA Guidelines for the Euthanasia of Animals. The animal protocol was approved by the Lawrence Berkeley National Laboratory Animal Welfare and Research Committee (AWRC).

## Author Contributions

WT, MA and RR conceived the project and interpreted the experiments and wrote the manuscript. WT performed experiments and analyzed the data. JS analyzed the data. HP, DM and KM provided experimental support.

## Conflict of Interest

The authors declare that the research was conducted in the absence of any commercial or financial relationships that could be construed as a potential conflict of interest. The reviewer DO declared a past co-authorship with one of the authors RR to the handling Editor.

## Footnotes

^1^https://www.avma.org/KB/Policies/Documents/euthanasia.pdf

## Supplementary Material

FIGURE S1 Line profile plots of electron tomographic densities. **(A–C)** Three examples of electron tomographic density map slices shown in non-orthogonal orientation. Top: EM density map and the boxed region of interest for line profile. Middle: corresponding line profile of the box-averaged line profile (to minimize high-frequency shot noise = pixel-to-pixel variation) showing distinct minima reflecting the dark density of the raw tomogram. Bottom: model superimposed onto the EM density map. Note vertical dashed lines depicting correlation of the position of the model rods with the distinct extrema, emphasizing that all models were built at locations of high density between either the actin and the SSC or the actin and the plasma membrane.

FIGURE S2 Model building and segmentation. **(A)** EM density map with clearly distinguishable actin-SSC connections between the SSC membrane and the actin filaments as well as the actin filaments and the Plasma Membrane (PM; pillars). **(B)** Simplified ball-and-stick model of SSC membrane (red), actin-SSC links (yellow), actin filaments (orange), pillars (green), and the PM (blue). **(C)** ORS Dragonfly super-pixel option segmented filaments. The segmented volumes suggest additional details compared to the simplified models. However, such details could have been altered by sample preparation and thus would first need to be verified by cryo-tomography imaging. On the other hand, the presence of a connection is not in doubt, a point we submit is best made by a simplified model **(D)**. The simplified ball-and-stick model can be readily examined for geometrical measurements **(E)**, such as pillar and actin-SSC link length (shown in **E**) or distribution (not shown).

## Abbreviations

OHC: Outer Hair Cell
SSC: Subsurface cisterna
ECS: Extracisternal space
CL: Cortical lattice
PM: Plasma Membrane
LM: Lumenal Material
HPF: High-pressure freezing
FS: Freeze-substitution.
