# Supplementary figures and images for: 3D Ultrastructure of the Cochlear Outer Hair Cell Lateral Wall Revealed By Electron Tomography
Source: Front Cell Neurosci. 2019 Dec 20;13:560. doi: 10.3389/fncel.2019.00560 (PMC6933316; doi:10.3389/fncel.2019.00560)

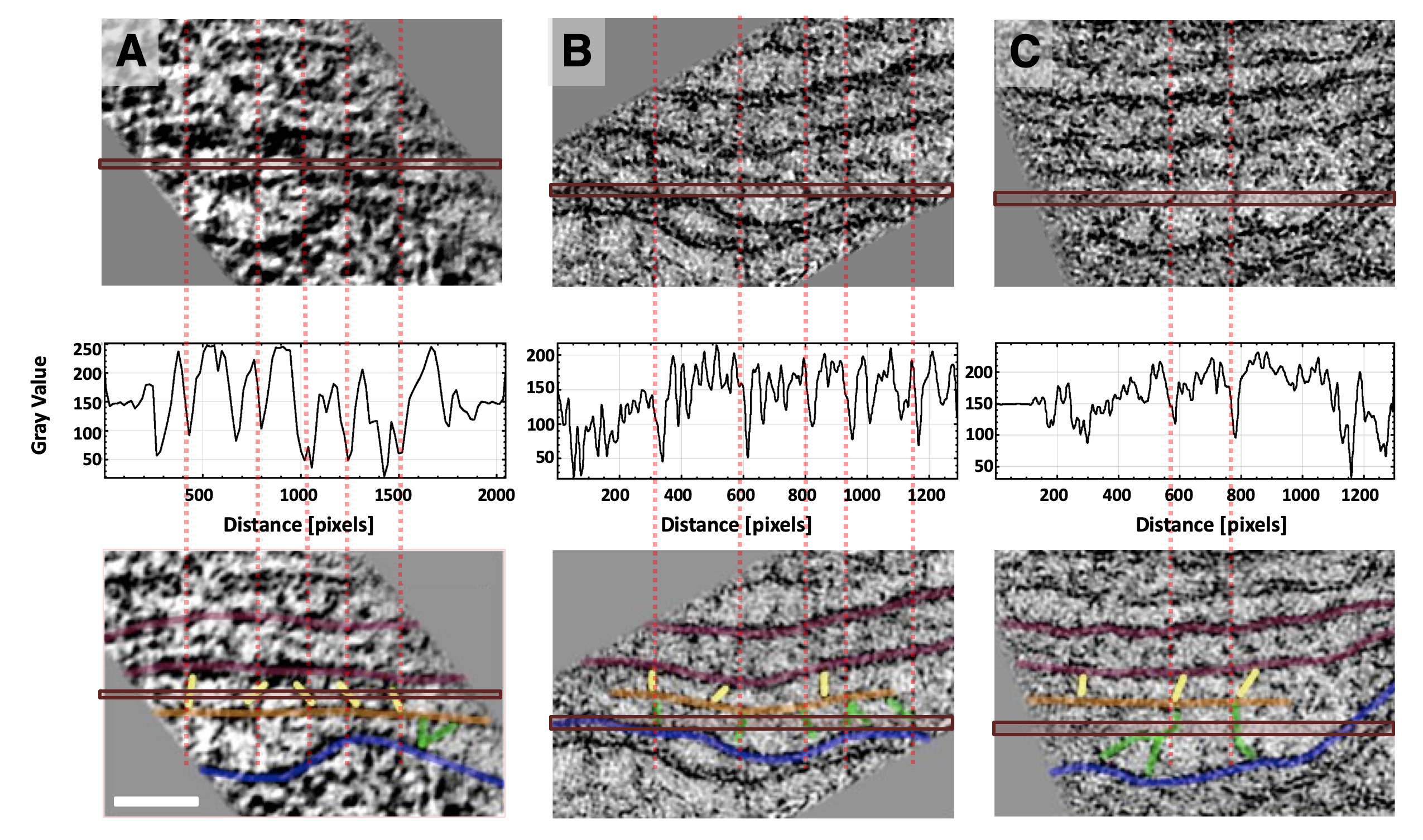

Supplement: FIGURE S1 — Line profile plots of electron tomographic densities. (A–C) Three examples of electron tomographic density map slices shown in non-orthogonal orientation. Top: EM density map and the boxed region of interest for line profile. Middle: corresponding line profile of the box-averaged line profile (to minimize high-frequency shot noise = pixel-to-pixel variation) showing distinct minima reflecting the dark density of the raw tomogram. Bottom: model superimposed onto the EM density map. Note vertical dashed lines depicting correlation of the position of the model rods with the distinct extrema, emphasizing that all models were built at locations of high density between either the actin and the SSC or the actin and the plasma membrane. [file Image_1.png]

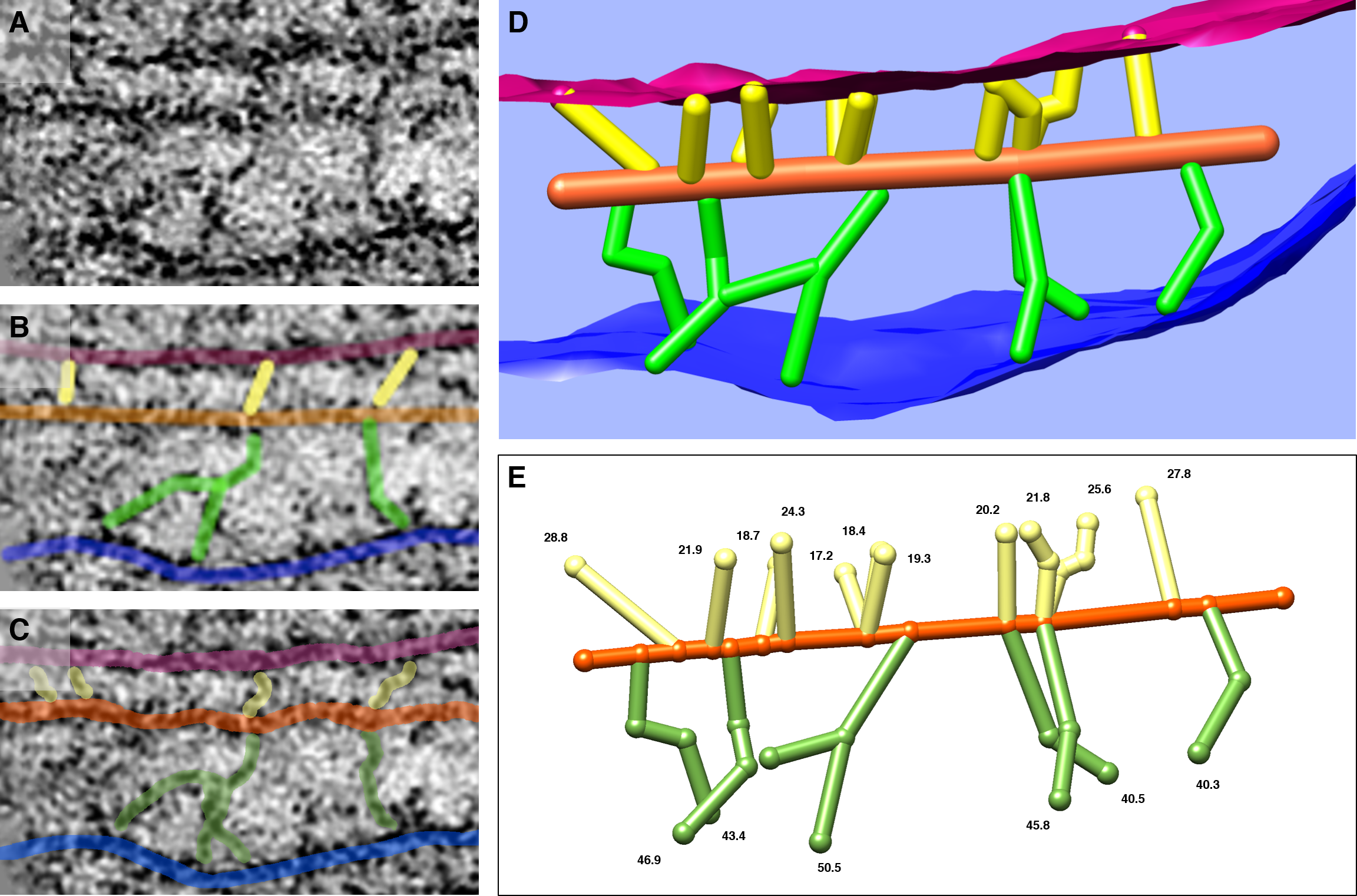

Supplement: FIGURE S2 — Model building and segmentation. (A) EM density map with clearly distinguishable actin-SSC connections between the SSC membrane and the actin filaments as well as the actin filaments and the Plasma Membrane (PM; pillars). (B) Simplified ball-and-stick model of SSC membrane (red), actin-SSC links (yellow), actin filaments (orange), pillars (green), and the PM (blue). (C) ORS Dragonfly super-pixel option segmented filaments. The segmented volumes suggest additional details compared to the simplified models. However, such details could have been altered by sample preparation and thus would first need to be verified by cryo-tomography imaging. On the other hand, the presence of a connection is not in doubt, a point we submit is best made by a simplified model (D). The simplified ball-and-stick model can be readily examined for geometrical measurements (E), such as pillar and actin-SSC link length (shown in E) or distribution (not shown). [file Image_2.TIF]
